# Risk factors for intraoperative hemorrhage of Type I neurofibromatosis

**DOI:** 10.1186/s12893-023-02067-7

**Published:** 2023-06-10

**Authors:** Qianqian Gao, Siwei Qu, Ning Ma, Weixin Wang, Sen Chen, Zhe Yang, Yangqun Li

**Affiliations:** 2nd Department, Plastic Surgery Hospital, Chinese Academy of Medical Sciences and Peking Union Medical College, No. 33 Badachu Road, Shijingshan District, 100144 Beijing, China

**Keywords:** Neurofibromatosis, Neurofibroma, Hemorrhage, Risk factors

## Abstract

**Introduction:**

Neurofibromatosis (NF) is an inherited disease and a benign tumor originating from nerve sheath cells. Neurofibromatosis type I (NF1) is the most common type, and most cases are characterized by neurofibromas. Neurofibromas in NF1 are mainly treated by surgery. Our study explores the risk factors for intraoperative hemorrhage in Type I neurofibromatosis patients who underwent neurofibroma resection.

**Methods:**

A cross-sectional comparison of the patients who had undergone resection of neurofibroma for NF1. Data regarding patient characteristics and data about operative outcomes were recorded. The definition of intraoperative hemorrhage group was the intraoperative blood loss greater than 200 ml.

**Results:**

Of 94 eligible patients, 44 patients were in the hemorrhage group and 50 patients were in the non-hemorrhage group. Multiple logistic regression analysis demonstrated that the area of excision, classification, surgical site, primary surgical, and organ deformation were significant independent predictors of hemorrhage.

**Conclusion:**

Early treatment can reduce the tumor cross-sectional area, avoid organ deformation, and reduce intraoperative blood loss. For plexiform neurofibroma or neurofibroma of the head and face, the amount of blood loss should be predicted correctly, and preoperative evaluation and blood preparation should be paid more attention to.

## Introduction

Neurofibromatosis (NF) is an inherited disease and a benign tumor originating from nerve sheath cells. It can occur in the brain, spinal cord and peripheral nerves [1–3]. Neurofibromatosis can be divided into 3 types: Neurofibromatosis type I (NF1), Neurofibromatosis type II (NF2) and schwannomatosis (SWN) [4]. Among the three types, NF1 is the most common type. NF1 is an autosomal dominant disease caused by mutations in the NF1 gene located at chromosome 17 [5]. Patients with NF1 are characterized by neurofibromas (peripheral nerve tumors), café-au-lait spots, skin-fold freckling, iris hamartomas, optic nerve gliomas, characteristic skeletal dysplasia of the long bones and sphenoid wing [6]. Additionally, NF1 patients also have multiple system involvement, with a high rate of deformity and disability. Therefore, NF1 can cause psychological disorders and reduce the quality of life in patients. Patients with NF1 have a 15-year shorter life expectancy than the general population [7]. The incidence of malignant tumors and vascular disease has been significantly higher in NF1 patients aged < 40 years [8]. It’s known that NF1 is one of the most challenging diseases to treat and neurofibromas in NF1 are mainly treated by surgery. Small tumors can be directly excised and sutured, and residual wounds after resection of large tumors can be covered by local flaps or skin grafts.

NF1 is caused by mutation of the NF1 gene responsible for the neurofibromin production. Neurofibromin is expressed in vascular endothelium and smooth muscle cells, which causes the vascular walls to thicken and the lumens to become smaller [9]. Moreover, aneurysm formation, high brittleness, and spontaneous rupture can occur. Neurofibroma constitution is brittle and abundant vascularity, and honeycomb vascular sinuses in the tumor. Therefore, it is difficult to stop bleeding and the amount of bleeding is large, which endangers the patient’s life [10]. One of the most important thing in neurofibroma resection is to reduce the amount of blood loss. Intraoperative tight hemostasis, postoperative compression bandage, the use of hemostatic drugs and other measures are used to reduce the amount of bleeding and reduce the rate of postoperative blood transfusion.

In this paper, the surgical information of NF1 in our department was retrospectively studied, and our study explores the risk factors for intraoperative hemorrhage of NF1 patients who underwent neurofibroma resection.

## Material and methods

A retrospective cohort study was done of all patients who had undergone resection of neurofibroma for NF1 at the Plastic Surgery Hospital from June 2015 to May 2022. Those who did not undergo surgical resection after admission were excluded. All operations were performed by LYQ and YZ. This study was approved by the Medical Ethics Board of the Plastic Surgery Hospital [2023(63)].

Included criteria of our study: NF1 was clinically diagnosed and confirmed by postoperative routine pathology; the patient had normal preoperative coagulation test results and had not taken or stopped anticoagulant or antiplatelet drugs for at least 1 week; the patient had signed informed consent for surgery. The surgical procedure was to remove the neurofibroma first, the direct suture was used for small wounds, local flaps were used to cover the wounds for large wounds, and skin grafting was performed for incomplete wounds with local flaps.

Data regarding patients’ characteristics, such as age, sex, weight, area of excision, operative time, hospital stay, estimated blood loss, blood transfusion, surgical site, neurofibroma classification, nationality, other symptoms and so on were recorded. The area of excision was defined as the product of the long diameter and the wide diameter of the neurofibroma. For multiple neurofibromas, the area of excision was the sum of the resected area of multiple neurofibromas. In addition, data about operative outcomes, including the area of excision, operation time, duration of hospital stay, intraoperative blood loss and need for transfusion were recorded from the chart. The definition of intraoperative hemorrhage group was the intraoperative blood loss greater than 200 ml.

### Statistical methods

All statistical analyses were performed using SPSS version 25.0 (IBM Corporation, Armonk, NY, USA). Descriptive statistics were used to summarize all characteristics and operative outcomes. The normality of continuous variables was tested using the Kolmogorov Smirnov test. The variables following normal distribution were analyzed by using the independent samples t-test, and the Mann Whitney U-test was used to compare the variables that did not follow a normal distribution. Disordered categorical variables were analyzed by using the Chi-squared test or Fisher’s exact test, and the Mann Whitney U-test was carried out to analyze the ordered categorical variables. A multivariate logistic regression analysis was then performed to identify risk factors for intraoperative hemorrhage based on factors associated with hemorrhage. Based on the combination of significant variables, we calculated the screening performance. All *P*-values were two-sided, and *P* < 0.05 was considered to be statistically significant.

## Results

A total of 105 patients were diagnosed with NF1 during the study period. Among these patients, 94 patients met the inclusion criteria. In Table 1, we present the demographic characteristics of the study population and the operative outcomes. The mean area of excision was 225.70 ± 750.43 cm^2^ and the mean operative time was 110.27 ± 56.56 min. The average number of hospitalization days was 10.02 ± 5.55 days, and the average intraoperative bleeding volume was 452.35 ± 1412.10 ml. The proportion of patients requiring transfusion was 8.5%. Removal of a single neurofibroma was done in 34.0% of included patients. There were 73 (77.7%) patients who underwent the initial operation.

**Table 1:** Demographics and operating outcomes of study population

Variable	*N* = 94
Age (years)	19.83 ± 10.62
Weight (kg)	51.67 ± 19.37
Area of excision(cm^2^)	225.70 ± 750.43
Operative time (min)	110.27 ± 56.56
Hospital stay (day)	10.02 ± 5.55
Estimated blood loss (ml)	452.35 ± 1412.10
Need for transfusion	8 (8.5)
Sex	
Female	48 (51.1)
Male	46 (48.9)
Surgical site	
Scalp and face or neck	70 (74.5)
Trunk and limbs	24 (25.5)
Classification	
Cutaneous	30 (31.9)
Plexiform	64 (68.1)
Nationality (Han)	85 (90.4)
Primary surgical	73 (77.7)
Single lesion	32 (34.0)
Other symptoms	
Café-au-lait spots	81 (86.2)
Organ deformation	60 (63.8)
Dyskinesia	22 (23.4)
Local pain	7 (7.5)
Ocular symptoms	17 (18.1)

All values are expressed as mean (± standard deviation) or number (%)

Operative hemorrhage occurred in 44 patients (46.8%). Other operative outcomes in cases with and without hemorrhage are shown in Table 2. Patients in the hemorrhage group had significantly more estimated blood loss, larger area of excision, more need for transfusion, and longer operative stay in hospital than patients in the non-hemorrhage group (*P* < 0.001, *P* < 0.001, *P* = 0.002, and *P* < 0.001, respectively).

**Table 2:** Comparison of operative outcomes in the hemorrhage and non-hemorrhage group

Parameters	Non-hemorrhage (*N* = 50)	Hemorrhage (*N* = 44)	*P* value
Estimated blood loss (ml)	32.5 (15–50)	400 (300–800)	< 0.001
Area of excision (cm^2^)	65 (30–150)	150 (81–265)	< 0.001
Operative time (min)	95 (70–120)	105 (81.25–138.75)	0.098
Hospital stay (day)	6.5 (5–8)	12.5 (8.25–16)	< 0.001
Need for transfusion	0 (0.0)	8 (18.2)	0.002

All values are expressed as median (interquartile range) or number (%)

Patient characteristics, including sex, nationality, primary surgical, café-au-lait spots, dyskinesia, local pain, or ocular symptoms showed no significant differences between the operative hemorrhage and non-hemorrhage groups (Table 3). There was no significant intergroup difference in the surgical site (Table 3). The median age in the operative hemorrhage group showed significantly older than that of the non-hemorrhage group (*P* < 0.001). There were significant differences in weight and classification between the operative hemorrhage and non-hemorrhage groups (*P* < 0.001, *P* < 0.001). Multiple logistic regression analysis demonstrated that the area of excision (odds ratio [OR], 1.008; 95% confidence interval [CI], 1.001–1.015, *P* = 0.021), classification (odds ratio [OR], 0.123; 95% confidence interval [CI], 0.027–0.570, *P* = 0.007), surgical site (odds ratio [OR], 16.089; 95% confidence interval [CI], 2.335–110.859, *P* = 0.005), primary surgical (odds ratio [OR], 5.099; 95% confidence interval [CI], 1.161–22.399, *P* = 0.031), organ deformation (odds ratio [OR], 0.176; 95% confidence interval [CI], 0.047–0.659, *P* = 0.01) were significant independent predictors of hemorrhage (Table 4). The ROC curves analysis showed that the area under the curve for the prediction of operative hemorrhage was 0.899 (95% CI, 0.837–0.961, *P* < 0.001).

**Table 3:** Comparison of demographic characteristics and parameters of neurofibroma in the hemorrhage and non-hemorrhage group

Variable	Non-hemorrhage (*N* = 50)	Hemorrhage (*N* = 44)	*P* value
Age (years)	16 (8.75–22.00)	21 (16.25–29.00)	< 0.001
Weight (kg)	48.04 ± 22.91	55.81 ± 13.43	< 0.001
Sex			0.295
Female	23 (46.0)	25 (56.8)	
Male	27 (54.0)	19 (43.2)	
Surgical site			0.158
Scalp and face or neck	34 (68.0)	36 (81.8)	
Trunk and limbs	16 (32.0)	8 (18.2)	
Classification			< 0.001
Cutaneous	24 (48.0)	6 (13.6)	
Plexiform	26 (52.0)	38 (86.4)	
Nationality (Han)	43 (86.0)	42 (95.5)	0.167
Primary surgical	36 (72.0)	37 (84.1)	0.160
Single lesion	22 (44.0)	10 (22.7)	0.030
Other symptoms			
Café-au-lait spots	40 (80.0)	41 (93.2)	0.065
Organ deformation	24 (48.0)	36 (81.8)	< 0.001
Dyskinesia	10 (20.0)	12 (27.3)	0.406
Local pain	2 (4.0)	5 (11.4)	0.246
Ocular symptoms	6 (12.0)	11 (25.0)	0.102

All values are expressed as mean (± standard deviation), median (interquartile range) or number (%)

Independent-Samples t test, Mann Whitney U-test, Chi-squared test or Fisher’s exact test was used as appropriate

**Table 4:** Multiple logistic regression analysis for the prediction of operative hemorrhage

Parameters	OR (95%)	*P* value
Age (years)	0.979 (0.903–1.062)	0.612
Weight (kg)	1.040 (0.990–1.092)	0.117
Area of excision (cm^2^)	1.008 (1.001–1.015)	0.021
Operative time (min)	1.002 (0.985–1.020)	0.797
Surgical site	16.089 (2.335–110.859)	0.005
Classification	0.123 (0.027–0.570)	0.007
Primary surgical	5.099 (1.161–22.399)	0.031
Single lesion	0.494 (0.135–1.807)	0.286
Other symptoms		
Organ deformation	0.176 (0.047–0.659)	0.010

*OR* Odds ratio, *95% CI* 95% Confidence interval

## Discussion

Neurofibromatosis is a dominant genetic disorder originating from nerve sheath cells. The neurofibromatosis disease spectrum includes neurofibromatosis types 1 and 2 (NF1, NF2) and schwannomatosis (SWN). Friedrich von Recklinghausen first described NF1 in 1882 as a systemic disorder [11]. NF1 is a genetic tumor predisposition syndrome associated with malformation, in which tumors grow in the nervous system and involve other organ systems [12]. The incidence is approximately 1 in 3000 births, regardless of gender or ethnicity. The diagnosis is mainly based on the clinical criteria established by the National Institutes of Health (NIH) at the consensus meeting of the year 1987 [13]. Presence of ≥ 2 of the following can be diagnosed as NF1: (1) With ≥ 6 café-au-lait spots: > 5 mm in diameter in prepubertal individuals and > 15 mm in postpubertal individuals; (2) With ≥ 2 neurofibromas of any type or 1 plexiform neurofibroma; (3) Freckling in the axillary or inguinal regions; (4) Optic glioma; (5) With ≥ 2 Lisch nodules; (6) A distinctive osseous lesion such as sphenoid wing dysplasia or thinning of long bone cortex, with or without pseudoarthrosis; (7) First-degree relative (parents, sibling, or offspring) with NF1 based on above criteria. Patients’ life expectancy is about 15 years shorter than normal, mainly due to malignancies, heart attacks and strokes [7, 12, 14].

There is a high incidence of cutaneous neurofibroma (CNF) among NF1 patients. In adolescence, cutaneous neurofibroma (CNF) usually appears, and they become more numerous in adulthood [14]. In addition, subcutaneous and plexiform neurofibromas (PNF) are seen in 30–50% of NF1 individuals. In the embryo, PNF is already present. In the beginning, they are benign and grow in a reticular pattern, replacing normal tissue. There is an approximate 8–13% chance that PNF will degenerate into malignant peripheral schwannoma in NF1 patients. Patients with NF1 tend to have lower life expectancy due to these early metastases of PNF [12, 15].

This study was divided into hemorrhage group and non-hemorrhage group according to the amount of intraoperative blood loss. There were statistical differences between the two groups in the area of neurofibroma resection, the length of hospital stay, and the need for blood transfusion. In some other types of surgery, increased blood loss can significantly increase the length of hospital stay [16, 17]. Our results also show that intraoperative blood loss can affect the length of hospital stay.

We compared demographic characteristics and parameters of neurofibroma between the hemorrhage and non-hemorrhage groups, and found statistical differences in age, weight, neurofibroma classification, single lesion, and accompanied organ deformation. Neurofibromas in most patients with NF1 may be visible on the skin’s surface or within the dermis in childhood, but the number or size of neurofibromas generally increases after puberty [11, 18]. Therefore, older patients may have larger neurofibromas, leading to greater intraoperative blood loss. We thought this reason can explain the statistical differences in age and weight between the two groups. Therefore, we suggest that patients with NF1 should visit the doctor as early as possible. If the neurofibromas are large or growing, it can be removed as early as possible to avoid its enlargement and increase the risk of surgical bleeding. Cutaneous neurofibromas are benign and arise in and are limited to the skin, while plexiform neurofibromas have malignant potential and show a clear association with myelinated nerves [19]. Because cutaneous neurofibromas are limited to the epidermis and dermis, blood supply is less abundant. Plexiform neurofibromas may develop into diffuse tumors that invade surrounding soft tissues and may be accompanied by vascular changes [20]. Plexiform neurofibromas have an abundant blood supply, and have vascular sinus, and bleeding during surgical resection. Plexiform neurofibromas had more intraoperative bleeding, which is consistent with our results. The organ deformations of patients in our study were often facial damage. The facial neurofibroma can occlude the eyeball or they are too large and drooping resulted in organ deformations. These patients with organ deformation were mostly caused by facial neurofibroma, with rich blood supply of the neurofibroma in the head and face, and were more prone to bleeding. Thus, there were more patients with organ deformation in the hemorrhage group.

This study showed that neurofibroma cross-sectional area was an independent risk factor affecting the amount of blood loss. In clinical work, surgeons mostly estimate the amount of neurofibroma blood loss by tumor size and cross-sectional area, and prepare blood before surgery. In other benign tumors, tumor size is also an independent risk factor for blood loss [21]. Therefore, surgery is more effective in reducing intraoperative bleeding when the neurofibroma is small. Classification, surgical site and organ deformation were also independent risk factors for increased intraoperative blood loss. The plexiform neurofibroma infiltrates the surrounding soft tissues with more abundant blood supply and numerous venous sinuses. The head and face have more blood supply than the trunk and extremities. The characteristics of typing and anatomy are consistent with our results. The neurofibroma with organ deformation was large, and the deformable organs were mostly in the head and face, which affected the amount of intraoperative blood loss. The primary operation is also an independent risk factor affecting the amount of blood loss, which may be due to the patient seeking medical care late, resulting in the size larger, thus affecting the amount of intraoperative blood loss.

The results of this study showed that neurofibroma cross-sectional area, classification, surgical site, primary operation and organ deformation were all independent risk factors affecting intraoperative blood loss. Early treatment can reduce the tumor cross-sectional area, avoid organ deformation, and reduce intraoperative blood loss. For plexiform neurofibroma or neurofibroma of the head and face, the amount of blood loss should be predicted correctly, preoperative evaluation and blood preparation should be paid more attention to, and accurate hemostasis should be performed during operation to reduce the amount of intraoperative blood loss.

## Data Availability

The datasets used and analyzed during the current study available from the corresponding author on reasonable request.
